# Factors affecting tuberculosis health message recall 2 years after active case finding in Blantyre, Malawi

**DOI:** 10.5588/ijtld.18.0006

**Published:** 2018-09

**Authors:** E. J. M. Monk, M. Kumwenda, M. Nliwasa, J. Mpunga, E. L. Corbett

**Affiliations:** * London School of Hygiene & Tropical Medicine, London, UK; † Malawi-Liverpool Wellcome Trust Clinical Research Programme, Blantyre; ‡ Department of Microbiology, College of Medicine, Blantyre; § National Tuberculosis Programme, Lilongwe, Malawi

**Keywords:** behaviour change, health seeking, Health Belief Model

## Abstract

**SETTING:**

Urban slums, Blantyre, Malawi.

**OBJECTIVE:**

To explore tuberculosis (TB) community-wide active case finding (cwACF) recall and accompanying messaging 2 years after the intervention.

**DESIGN:**

This mixed-methods study used population-weighted random cluster sampling to select three cwACF-receiving and three non-cwACF-receiving neighbourhoods in Blantyre. Qualitative data were collected using 12 focus group discussions (community peer-group members) and five in-depth interviews (TB officers) with script guides based on the concepts of the Health Belief Model (HBM). Thematic analysis was used to explore transcripts employing deductive coding. Questionnaires completed by focus group participants were used to collect quantitative data, providing a ‘knowledge score’ evaluated through univariate/multivariate analysis, analysis of variance and multiple linear regression.

**RESULTS:**

Community peer-group participants (*n* = 118) retained high awareness and positive opinions of cwACF and recognised the relationship between early diagnosis and reduced transmission, considering cwACF to have prompted subsequent health-seeking behaviour. TB-affected individuals (personal/family: 47.5%) had significantly higher knowledge scores than unaffected individuals (*P* = 0.039), but only if resident in cwACF-receiving neighbourhoods (*P* = 0.005 vs. *P* = 0.582), implying effect modification between exposures, albeit statistically under-powered (*P* = 0.229).

**CONCLUSION:**

Consistent with epidemiological evidence and HBM theory, cwACF may have a permanent impact on knowledge and behaviour, particularly in communities with a high prevalence of TB-affected individuals. Behaviour change strategies should be explicitly included in cwACF planning and evaluation.

## Abstract (FR)

**CONTEXTE:**

Bidonvilles urbains, Blantyre, Malawi.

**OBJECTIF:**

Explorer la mémorisation de la recherche active des cas de tuberculose (TB) dans toute une communauté (cwACF) et des messages qui l'ont accompagné 2 ans après l'intervention.

**SCHÉMA:**

Cette étude à méthodes mixtes a utilisé un échantillonnage aléatoire en grappes pondéré sur la population afin de sélectionner trois quartiers bénéficiant de cwACF et trois quartiers n'en bénéficiant pas à Blantyre, Malawi. Douze discussions en groupe focal (groupes de pairs membres de la communauté) et cinq entretiens approfondis (personnel TB) ont recueilli des données qualitatives avec des guides d'entretien calqués sur les concepts du Modèle des valeurs de la santé (HBM). Une analyse thématique a exploré les transcriptions, grâce à un codage déductif. Les questionnaires remplis par les participants des groupes focaux ont recueilli des données quantitatives, fournissant un « score de connaissance » évalué par analyse univariée/multivariée, analyse de la variance et régression multiple linéaire.

**RÉSULTAT:**

Les participants des groupes de pairs de la communauté (*n* = 118) ont montré un niveau élevé de sensibilisation et des opinions positives des cwACF, et ont reconnu la relation entre diagnostic précoce et réduction de la transmission, considérant que la cwACF amenait ensuite à une recherche de soins de santé. Les personnes affectées par la TB (à titre personnel/familial, 47,5%) ont eu des scores de connaissance significativement meilleurs que les personnes non affectées (*P* = 0,039), mais seulement si elles habitaient dans des quartiers bénéficiant de cwACF (*P* = 0,005 contre *P* = 0,582), ce qui implique une modification de l'effet en fonction de l'exposition, bien que statistiquement peu puissant (*P* = 0,229).

**CONCLUSIO:**

En accord avec les preuves épidémiologiques et la théorie HBM, la cwACF peut avoir un impact durable sur les connaissances et les comportements, particuli èrement au sein des communautés ayant une prévalence élevée de personnes affectées par la TB. Des stratégies de changement de comportement devraient être explicitement incluses dans la planification et l'évaluation de la cwACF.

## Abstract (ES)

**MARCO DE REFERENCIA:**

Tugurios urbanos de Blantyre, en Malawi.

**OBJETIVO:**

Explorar la memoria de una operación de búsqueda activa (cwACF) de casos de tuberculosis (TB) en toda la comunidad y los mensajes retenidos 2 años después de la intervención.

**MÉTODO:**

En el presente estudio de métodos mixtos se utilizó un muestreo poblacional con aleatorización por conglomerados ponderados, con el fin de escoger en Blantyre tres comunidades que habían participado en la cwACF y tres comunidades donde no se había ejecutado. Se recogieron datos cualitativos en 12 sesiones de grupos de opinión (con grupos de pares de miembros de la comunidad) y cinco entrevistas exhaustivas (con profesionales encargados de la TB) en las cuales se utilizaron guiones de orientación según los conceptos del Modelo de Creencias de Salud (HBM). Las transcripciones se evaluaron mediante análisis temático con una codificación deductiva. Los cuestionarios completados por los participantes en las sesiones de grupo recogían datos cuantitativos que daban una ‘puntuación de los conocimientos’ y se evaluaron por medio de análisis univariantes o multivariantes, análisis de la varianza y regresión lineal múltiple.

**RESULTADOS:**

Los participantes en los grupos de pares de la comunidad (*n*=118) habían conservado una gran sensibilización y opiniones positivas sobre la cwACF, reconocieron la relación entre el diagnóstico temprano y la disminución de la transmisión de la TB y afirmaron que la campaña estimuló sus comportamientos ulteriores de búsqueda de atención sanitaria. Las personas afectadas por la TB (personal o familiar 47,5%) obtuvieron puntuaciones de conocimientos notablemente má s altas que las personas no afectadas (*P* = 0,039), pero solo cuando residían en comunidades que habían participado en la cwACF (*P*=0,005 contra *P* = 0,582), lo cual sugiere una modificación del efecto según el tipo de exposición, pero con baja potencia estadística (*P* = 0,229).

**CONCLUSIÓN:**

En concordancia con la evidencia epidemiológica y la teoría HBM, la cwACF de TB en la comunidad puede tener una repercusión duradera sobre los conocimientos y los comportamientos, sobre todo en las comunidades con una alta prevalencia de personas afectadas por la TB. Es necesario incluir de manera explícita las estrategias de modificación del comportamiento en la planificación y la evaluación de cwACF.

TUBERCULOSIS (TB) is the leading cause of adult infectious deaths globally, with 1.7 million deaths in 2016. The World Health Organization's (WHO's) End TB Strategy now emphasises global TB elimination.^1^ Sub-Saharan Africa has the highest case notification rates (CNRs) and per capita mortality due to TB of any global region, but also the lowest case detection rates (CDRs).^2^ Recent African national TB prevalence surveys, the first in many decades, have shown a several-fold higher prevalence of undiagnosed TB than anticipated in most countries, leading to an upward revision of global TB estimates.

Active case finding (ACF) is a component of the End TB Strategy and, as supported by mathematical modelling, is of high relevance to the increasing number of urban slums in Africa and Asia that combine a high prevalence of undiagnosed TB with low rates of case detection.^3^ The goal of ACF is to provide earlier case detection and treatment to accelerate reductions in undiagnosed TB and hence TB transmission.^4^,^5^

In Zimbabwe, undiagnosed culture-positive TB fell by 43% following six rounds of community-wide ACF (cwACF) in high-density residential neighbourhoods using 6-monthly door-to-door chronic cough enquiry, investigated using smear microscopy.^6^ Introducing a similar approach in Blantyre, Malawi, between 2011 and 2014 led to a pronounced and sustained effect on TB case notifications, with an 84% increase in adult smear-positive CNR (from 220 per 100 000 population to 405/100 000).^7^,^8^ Male sex, human immunodeficiency virus (HIV) positive status and older age were most strongly associated with higher adjusted ratios; CNR among males aged 30–39 years increased from 360/100 000 to 638/100 000 (*P* ⩽ 0.001). There was no corresponding change in non-cwACF comparator areas. However, direct diagnosis of smear-positive TB in the community was not sufficient to explain the magnitude of these changes, suggesting behavioural change through health promotion as the primary mechanism of action.

The main aims of the present study were to investigate the extent of cwACF recall 2 years after the intervention, compare TB knowledge, attitudes and health-seeking practices by place of residence (cwACF or non-cwACF neighbourhoods) and to explore the possible mechanisms for continued CNR improvement. We used the Health Belief Model (HBM), developed initially to support American TB ACF interventions in the 1950s, as our conceptual framework for study design.^9^,^10^ Each of the six HBM concepts has been associated with improved compliance with contact tracing and engagement with community screening programmes for TB, and has led to an increase in direct diagnostic yield and higher medication adherence.^11–14^ However, to our knowledge, this was the first study to explore the inverse relationship: how cwACF may feed back to HBM concepts and so potentially influence future health-seeking behaviour.

## STUDY POPULATION AND METHODS

### Study design

A mixed-methods research design was employed in this study; script-guides and questionnaires were designed using the HMB as a conceptual framework. Qualitative data provided deeper insights into participants' recollection of TB cwACF activities and accompanying messaging. Twelve community peer-group focus group discussions (FGDs) and five TB officer in-depth interviews (IDIs) were conducted in total; these were spread equally across three cwACF suburbs (Chilomoni, Likhubula and Ndirande) that received cwACF between 2011 and 2014, and three non-cwACF comparator suburbs (Bangwe, Machinjiri and Manase) with city-centre proximity and socioeconomic characteristics that were similar to the cwACF suburbs selected. The FGDs were vital in establishing the broader community knowledge of cwACF and linked messages about TB, while the IDIs provided deeper perspectives on patients' understanding of TB obtained from the accounts of TB officers through their cumulative engagement with clients being investigated for TB or receiving anti-tuberculosis treatment. Experienced researchers performed FGDs in Chichewa, the most popular language in the study setting, and IDIs in English. All FGDs and IDIs were audio-recorded using encrypted digital recorders.

A questionnaire was administered among community peer-group members before FGDs for quantitative analysis and triangulation with FGD and IDI data. This design allowed not only the capture of individual sociodemographics, but also an objective assessment of TB knowledge. Illiterate participants were assisted with one-to-one facilitation.

### Cluster selection

A two-stage population-weighed random cluster sampling approach was used. The initial sampling frame covered 315 recently enumerated community health worker (CHW) catchment areas, defined using Global Positioning System (GPS) coordinates captured using circumferential walks. Catchment areas considered unsuitable due to separate community-level intervention (HIV self-testing: 51 CHW catchment areas) or population density <10 000/km^2^ (eight CHW catchment areas) were excluded.

Population-weighted cluster sampling, stratified by cwACF/non-cwACF areas, was then used to select six CHW catchment area clusters; three cwACF clusters (Mulunguzi Sosola, Likhubula A and Majiga 1) and three non-cwACF clusters (Mbayani, Mkwate and Manase City).

### Community peer groups and participant selection

Community engagement was established using traditional authorities (‘chiefs’) and CHWs as point of entry. Formative community mapping and situational analysis were conducted in each selected cluster to identify active peer groups (e.g., sports, church, microfinance), providing individuals already known to one another for more informative FGDs. This also ensured homogeneity within each group to capitalise on shared experiences.^15^

Peer groups with a health-related theme were excluded to minimise background health education differences. As most peer groups were exclusively female, we stratified selection to provide six female-only and six mixed-sex peer groups using purposeful selection of two peer groups per selected cluster. All participants were purposively selected using their membership of eligible peer groups as an inclusion criterion.^16^ A questionnaire was administered to peer-group members immediately before conducting each FGD. The five TB officers serving the six selected clusters (one TB officer worked in two clusters) were also purposively approached for IDI based on their respective positions.

### Sample size

Twelve FGDs and five IDIs were estimated to be appropriate for information saturation, as indicated by data replication and category development for abstraction and interpretation.^17^ We considered a minimum of six participants for an FGD to proceed, preferably with 10–12 participants.

### Analytical methods

Audio-recorded qualitative data were transcribed and translated verbatim. Processed qualitative transcripts were then indexed and coded using NVivo 11.0 (QSR International, Melbourne, VIC, Australia). A thematic approach was used to analyse qualitative data with deductive coding to dissect and group data into meaningful segments. This scheme was amended throughout data coding and analysis to allow commonly emerging patterns within multiple themes to be categorised into a broader theme once sufficiently differentiated. Coded material was read several times and reconnected or grouped into broader themes using an iterative process.

Quantitative data were captured using TeleForm 10.7 (Hewlett Packard, Palo Alto, CA, USA). Responses to 10 true/false/don't know questions assessing TB knowledge were coded ‘1’ for a correct response and ‘0’ for an incorrect or ‘don't know’ response. Summative knowledge scores were analysed using univariate/multivariate analysis for group differences, and analysis of variance for trend with age and multiple linear regression for assessing effect modification. Normal distribution was confirmed using the Shapiro-Wilk test. Trends noted following quantitative analysis of the data were used for triangulation with FGD and IDI themes to validate the results, facilitate deeper understanding and identify consistency or convergence of emerging findings from the three different data sources used.^18^

### Ethical considerations

Ethical approval was granted by the College of Medicine Research Ethics Committee, University of Malawi, Blantyre, and the London School of Hygiene & Tropical Medicine, London, UK. Written informed consent (or witnessed thumbprint if illiterate) was provided by all participants; information sheets were available in both English and Chichewa.

## RESULTS

A total of 118 participants (58 from cwACF clusters, 102 women) were recruited for 12 FGDs. All participants completed a questionnaire before starting discussions. The median age was 37 years (interquartile range 30–47); 55 (46.8%) had secondary schooling or above, and 56 (47.5%) had experienced TB, either themselves or within their family (‘TB-affected’) (Table 1).

**Table 1:**
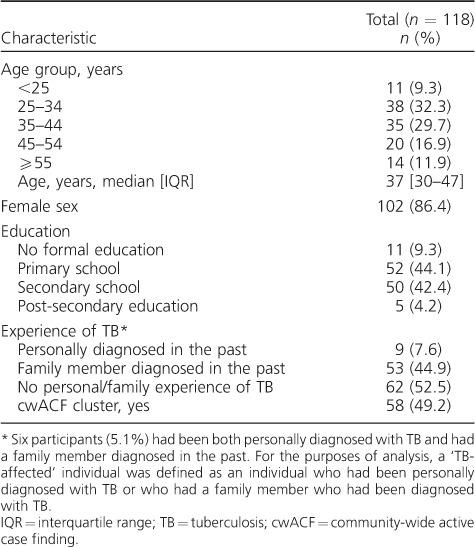
Baseline characteristics of participants

### Community memory of community-wide active case finding

Thematic analysis of FGD and IDI content indicated widespread community awareness of the cwACF programme in both intervention and comparator suburbs (Table 2, theme 6), including anticipation of screening arrival on a 6-monthly basis in cwACF areas during the intervention period (Table 2, quote 6.5). Community members and TB officers reported a beneficial impact of cwACF, which emerged as a theme of positive change (Table 2, theme 7). A strong agreement that there had been good community screening programmes in Blantyre was evident (‘strongly agree’ in 84.8% of questionnaire participants), with newly perceived benefits of screening (Table 2, quote 7.3) and appreciation of TB curability (Table 2, quote 7.1). There was evidence of change in personal health-seeking behaviour among FGD participants (Table 2, quote 7.4) and encouragement of community members with symptoms to self-present for testing (Table 2, quotes 7.2 and 7.4). Persistent behaviour change after the intervention had ceased was also reported, with subsequent self-presenting patients referring to messages they had learnt through the cwACF intervention (Table 2, quote 6.6).

**Table 2:**
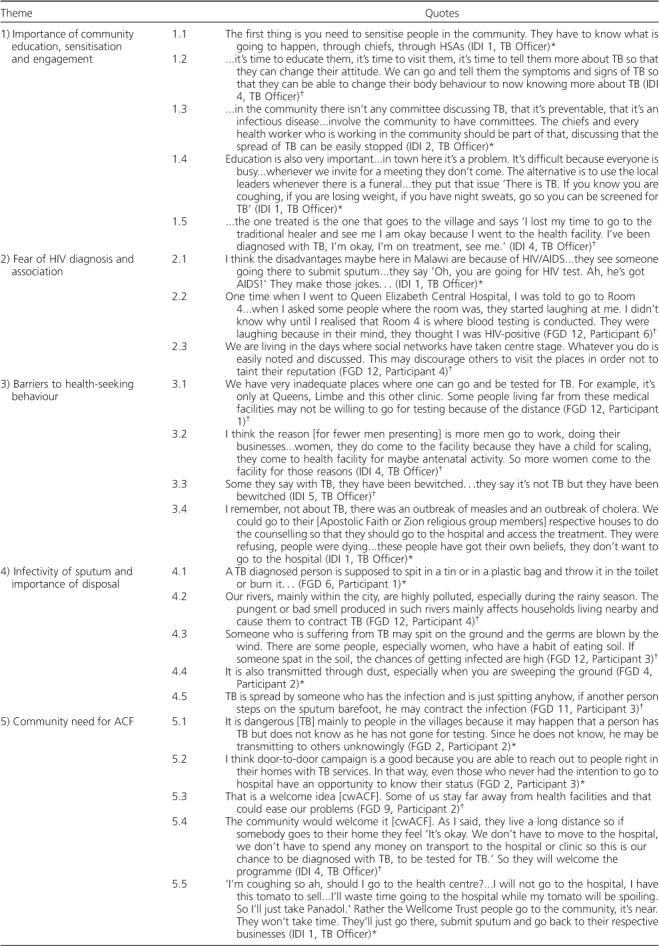

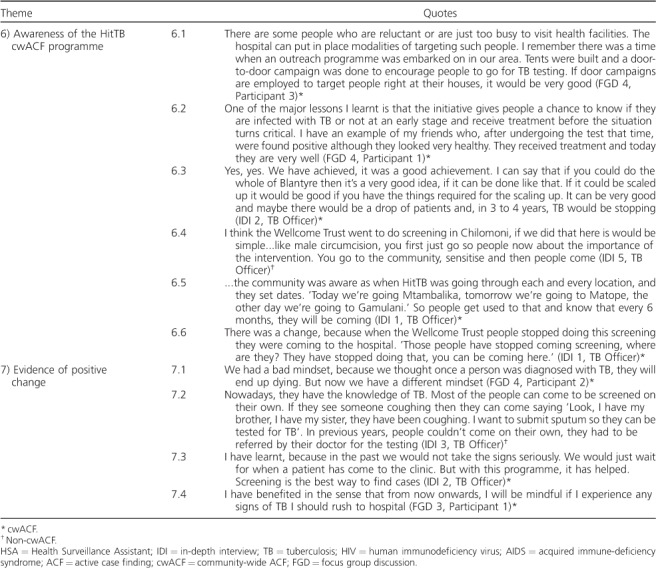
Themes and quotes from qualitative analysis

Detailed understanding of the messages provided during cwACF was more comprehensive in cwACF clusters: FGD participants affirmed the benefits of bringing screening to the doors of populations with poor access to centralised health care (Table 2, quotes 5.1 and 5.2). Participants also recognised the relationship between early diagnosis and reduced transmission (Table 2, quote 6.2); these insights were supported by personal anecdotes recounting interactions with the cwACF team (Table 2, quotes 6.1 and 6.2). Participants from non-cwACF cluster FGDs were able to identify populations within their community that would benefit from future cwACF programmes (Table 2, quote 5.3), a prospect welcomed by TB officers throughout Blantyre (Table 2, quotes 5.4, 6.3 and 6.4). There was high acceptability of cwACF, with 97.5% of participants strongly agreeing that they would engage with community teams offering free TB tests and 98.3% strongly agreeing that TB screening was important.

### The role of community mobilisation

The necessity of ongoing community engagement for the success of cwACF programmes was a recurrent theme from both FGDs and IDIs, with calls to sensitise community members to cwACF before implementation (Table 2, quote 1.1) and bring TB education to the community (Table 2, quote 1.2). One suggestion was to use survivors of TB to advocate for more appropriate health-seeking behaviour (Table 2, quote 1.5). Chief and Health Surveillance Assistant (HSA) engagement was recognised by TB officers as crucial during community mobilisation: without their involvement, community meetings appeared to have a low attendance of community members (Table 2, quote 1.3). A further suggestion was to use funerals as a platform for community TB education, as they are widely attended community events (Table 2, quote 1.4).

### Community knowledge of tuberculosis and personal experience

Knowledge of TB in both cwACF and non-cwACF areas was high: 99.2% reported TB to be curable and 90.6% believed that a 2-week cough should be investigated. Cough was recognised as the main transmission route by 96.6% of participants and weight loss or night sweating was identified as a symptom by 76.3%. There was no significant difference in TB knowledge score by age (*P* = 0.21), sex (*P* = 0.80) or cwACF exposure (*P* = 0.613). Personal experience of TB, reported by 47.5% of participants, was significantly associated with higher knowledge scores (*P* = 0.039) (Table 3).

**Table 3:**
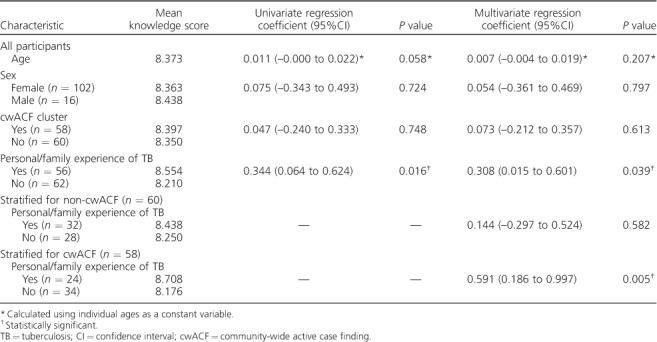
Analysis of differences in knowledge depending on age, sex, cluster type and personal/family experience of TB

On stratified analysis, the association between TB knowledge and personal TB experience was only apparent in cwACF areas, raising the possibility of effect modification. In cwACF areas, TB-affected individuals had significantly higher knowledge of TB than community members without personal TB experience (*P* = 0.005). This association was not present in non-cwACF areas (*P* = 0.582). Given the biological plausibility of learning potentiation between cwACF exposure and personal TB experience, we explored effect modification using multiple linear regression (*P* = 0.229).

### Misperceptions of sputum infectivity

Appropriate knowledge that TB can be tested using sputum collection was held by 98.3% of participants. However, misunderstanding over the nature of transmission and the extent of sputum infectivity was a strong theme emerging across all clusters. Sputum hygiene appeared to be considered an essential method of controlling TB, to the extent of being collected in sealed containers and disposed of in pit latrines or by burning (Table 2, quote 4.1). Inappropriate disposal was considered greatly worrying by members of the community, with the view that if patients were to spit on the floor, then TB would be contracted easily through eating soil, sweeping dust or walking barefoot (Table 2, quotes 4.3, 4.4 and 4.5). The odour due to river pollution was also believed to facilitate TB transmission (Table 2, quote 4.2).

### Belief in witchcraft and role of traditional healers

Most participants (55.9%), and particularly those with secondary school education or above (72.7%), strongly disagreed with the suggestion that traditional medicine was equal to or better than modern medication prescribed by a medical doctor for anti-tuberculosis treatment. Nonetheless, our discussants affirmed that bewitchment was a genuine entity and a common cause of serious illness that could include TB-like symptoms. As such, consultation with traditional healers following the manifestation of symptoms suggestive of TB was said to be a common cause of treatment delay (Table 2, quote 3.3).

### Stigma as a barrier to health care seeking

There was evidence of TB stigmatisation, with most community members strongly agreeing that they would want a family member's TB diagnosis to remain undisclosed. This may be a consequence of known TB and HIV association and heightened HIV stigma in communities (Table 2, quote 2.1). Stigmatising attitudes and perceptions increased with age in questionnaire responses: older age groups associated TB with dirtiness or uncleanliness and anticipated that they would be assumed to be HIV-positive if diagnosed with TB. Higher education levels were associated with reduced stigma across all questions addressing these topics. Community ‘gossip’, prompted by attending hospital-based health care, was a major concern (Table 2, quote 2.2), with the availability of social media increasing worries about the ease of rumours spreading (Table 2, quote 2.3).

### Further barriers affecting health-seeking behaviour

Women were thought by TB officers to have greater access to routine health care services than men, given their relative familiarity with clinics for antenatal/child care visits and more flexible schedules (Table 2, quote 3.2). Daily work pressure was believed to prevent access to these routine services among men. Other recognised barriers included large distances to TB clinics, with discussion around time limitation and the opportunity costs of visiting centrally based facilities (Table 2, quotes 3.1 and 5.5). Some barriers, notably religious objections, were considered absolute by TB officers, and therefore not amenable to change by cwACF (Table 2, quote 3.4).

## DISCUSSION

Our study found that there was a lasting recall of a door-to-door cwACF intervention 2 years after community-based activities had ceased. Both community residents and health workers (TB officers) viewed cwACF positively, describing many perceived benefits, some of which were said to be ongoing. Just under half of our participants reported close firsthand experience of TB, either personally or through a family member with TB. Intimate experience of TB was associated with significantly greater TB knowledge in community members living in areas that had received cwACF and accompanying health messages, but not in residents of non-intervention comparator areas. Finally, we identified widespread misperceptions relating to sputum infectivity and potentially originating from a misinterpretation of cwACF messages. These findings collectively suggest that cwACF, a widely applied public health intervention from the last century, provides highly effective community engagement, leading to prolonged retention of key TB messages and social diffusion, in addition to direct TB screening benefits. Health promotion strategies, ideally using contemporary multilevel strategies aimed at maximising and directing behaviour change, should be an integral part of planning cwACF campaigns. High engagement with TB messages by the many individuals personally affected by TB makes this subgroup an important resource and target audience.

Recall of TB messages and TB knowledge across Blantyre was substantially higher than expected based on knowledge, attitude and perception surveys in other African settings.^19–21^ Widespread community recall of not only the implementation of cwACF, but also its key health messages, was evident 2 years after the intervention had ceased. The cwACF intervention was delivered with a 6-monthly home enquiry for chronic cough, supported by leafleting and megaphone announcements in three large suburbs that are home to one third of the population of Blantyre, including the city's main market area. Evidence of recall of the intervention by Blantyre residents living in non-cwACF areas could then reflect either direct exposure to the cwACF team while visiting cwACF areas for work or other reasons, or social diffusion resulting from discussions about cwACF. In either case, this finding adds to the impression of cwACF as being highly memorable.

TB knowledge was significantly higher in community members who had personal/close family experience of TB than those without such experience, as previously described in a study from Asia.^22^ A novel finding in our study, however, was the extent to which this association was restricted to residents of cwACF areas, potentially reflecting the combination of 1) greater cumulative exposure to TB messages from the cwACF team and 2) greater engagement (and hence recall) by individuals personally affected by TB. Although formal testing for effect modification failed to show a statistically significant interaction, the current study was not powered to provide such evidence post-stratification: ‘absence of evidence’ rather than ‘evidence of absence’.^23^

Glanz et al.'s HBM, used as the basic conceptual framework for this study, is shown in the Figure. First developed to understand non-participation in American TB case-finding programmes of the 1950s, HBM is a motivational theory focused on individual-level factors and is still considered relevant by screening programmes today. The model assumes healthy behaviour to be a function of both individual knowledge and motivation, with motivation influenced by perceived vulnerability to and severity of the condition, as well as the likely benefits of and barriers to taking the recommended action steps. Interventions such as community screening, combined with appropriate health information addressing risks and benefits, provide specific ‘cues to action’. The importance of personal effort and resilience was added as perceived ‘self-efficacy’ in the 1970s.^24^

**Figure:**
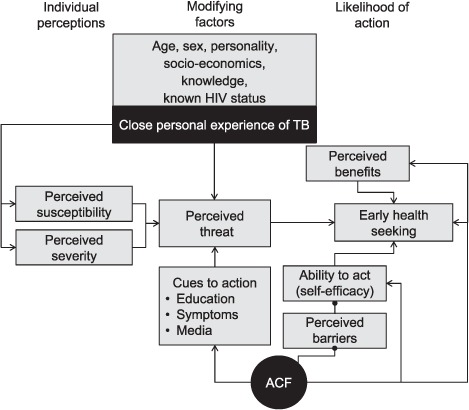
The Health Belief Model showing the likely roles of close personal experience of TB and cwACF on health seeking for cough. The starting point of the Glanz et al.'s Health Belief Model, including self-efficacy, is in light grey. Study findings are shown in black, and relate close personal experience of TB to perceived susceptibility, severity and threat, and mapping the multiple influences of exposure to cwACF on subsequent health-seeking behaviour as related by participants. TB = tuberculosis; HIV = human immunodeficiency virus; ACF = active case finding; cwACF = community-wide ACF.

Both close personal experience of TB and the periodic nature of cwACF, which offers multiple opportunities to be screened and to receive information, can be mapped to this framework, as shown in the Figure. Our participants considered cwACF as providing an immediate cue to action, an opportunity to effectively engage and actively acquire knowledge,^9^,^10^,^25^ a way to bypass facility access barriers and to demystify the diagnostic process. Our participants also described their recall of cwACF interactions during any subsequent episodes of cough, resulting in more prompt health seeking from facility-based services. This view was supported by the observations of TB officers. Suggestions that would reinforce both perceived susceptibility and severity within the community were offered spontaneously by TB officers, supporting this interpretation of the HBM. Public testimonial by former TB patients or dissemination of information about TB signs and symptoms at funerals, for example, may lead to improved health care engagement by providing an alternative to personal TB experience within one's own family for otherwise unaffected community members.

Another notable feature was how favourably the cwACF intervention was viewed, with no negative opinions voiced by any community or TB officer participant. A systematic review of the acceptability of community-based TB screening identified very high participation, with TB screening generally welcomed by communities despite the anticipated stigma when TB is diagnosed.^26^ From this position of trust, ACF services should aim for meticulous quality control to minimise harm, such as the expense, worry and stigma incurred by false-positive screening or confirmatory results. Ongoing behaviours related to the infectiousness of sputum, for example burying tins of collected sputum, would be a further example of the unintended consequences of cwACF if they result from misinterpretation of messages.

The main limitations of the study relate to our small sample size, reflecting the predominantly qualitative focus of this study, and the semi-purposive nature of recruitment (randomly selected active peer-group members), which was to ensure informative FGDs. Randomised cluster sampling was used, but with a second sampling unit (peer groups) that led to under-representation of men; sex has been reported to influence the HBM configuration, with female sex predicting a greater engagement with TB health messages.^27^ The HBM also has an expressly limited focus and is best considered as one component of a broader complex intervention strategy.^25^,^28–30^ For example, the HBM cannot adequately account for the changing social norms or other social dynamics observed during cwACF, such as social diffusion and collective encouragement.^31^ However, having been developed initially to support TB screening programmes, this framework provides a convenient and relevant starting point from which to add complexity to cwACF.

## CONCLUSION

Our mixed-methods study provides evidence of prolonged recall and behaviour change 2 years after the end of a cwACF programme, consistent with observed effects on TB CNRs that could not be explained by direct cwACF diagnosis alone. Close (personal or family) experience of TB was extremely common in this urban community with high HIV prevalence and TB incidence, and facilitated accurate retention and recall of cwACF messages. Programmes intending to include cwACF as part of their End TB Strategy should be aware of the high value of health promotion delivered during this type of intervention, ideally including behaviour change as an important and explicit end product to be optimised and included in all planning and evaluation stages.
